# Diagnosis and detection of pneumonia using weak-label based on X-ray images: a multi-center study

**DOI:** 10.1186/s12880-023-01174-4

**Published:** 2023-12-12

**Authors:** Kairou Guo, Jiangbo Cheng, Kaiyuan Li, Lanhui Wang, Yadong Lv, Desen Cao

**Affiliations:** Department of Biomedical Engineering, Chinese PLA General Hospital, Beijing, 100853 P.R. China

**Keywords:** Deep learning, Pneumonia, Detection and location, CAM, Weak supervision

## Abstract

**Purpose:**

Development and assessment the deep learning weakly supervised algorithm for the classification and detection pneumonia via X-ray.

**Methods:**

This retrospective study analyzed two publicly available dataset that contain X-ray images of pneumonia cases and normal cases. The first dataset from Guangzhou Women and Children’s Medical Center. It contains a total of 5,856 X-ray images, which are divided into training, validation, and test sets with 8:1:1 ratio for algorithm training and testing. The deep learning algorithm ResNet34 was employed to build diagnostic model. And the second public dataset were collated by researchers from Qatar University and the University of Dhaka along with collaborators from Pakistan and Malaysia and some medical doctors. A total of 1,300 images of COVID-19 positive cases, 1,300 normal images and 1,300 images of viral pneumonia for external validation. Class activation map (CAM) were used to location the pneumonia lesions.

**Results:**

The ResNet34 model for pneumonia detection achieved an AUC of 0.9949 [0.9910–0.9981] (with an accuracy of 98.29% a sensitivity of 99.29% and a specificity of 95.57%) in the test dataset. And for external validation dataset, the model obtained an AUC of 0.9835[0.9806–0.9864] (with an accuracy of 94.62%, a sensitivity of 92.35% and a specificity of 99.15%). Moreover, the CAM can accurately locate the pneumonia area.

**Conclusion:**

The deep learning algorithm can accurately detect pneumonia and locate the pneumonia area based on weak supervision information, which can provide potential value for helping radiologists to improve their accuracy of detection pneumonia patients through X-ray images.

## Key points

1.  Two publicly available datasets were used for model training and validation, and achieved very good pneumonia detection performance on independent external public data.
2.  The DL model based on weak supervision information that can accurately locate the pneumonia area.
3.  There is no COVID-19 pneumonia data in the training data, but the DL model can effectively detect COVID-19 pneumonia in independent external public data.

## Introduction

Pneumonia is a symptom of inflammation of the lungs, and can be caused by bacteria, viruses, or fungi. It accounts for more than 15% of deaths in children under 5-year [1, 2]. Especially in developing and underdeveloped countries, pneumonia are more likely to occur due to severe environmental pollution, unsanitary living conditions and inadequate medical infrastructure [3]. And pneumonia could be extremely dangerous and life-threatening if not be detected in the early stages [4]. Therefore, early diagnosis and interventional management are extremely important for patients with pneumonia. It can prevent the disease from becoming fatal.

And in clinical practice for screening pneumonia, chest X-ray imaging is the most commonly used method for diagnosing pneumonia. Because it is fast, low-invasive, low-cost, and simple to implement, X-ray imaging has become the standard method of screening pneumonia [5, 6]. However, the chest X-ray examinations for pneumonia screening is challenging. Radiologists with different experience may miss and misdiagnose due to subjective variability [7, 8]. Therefore, there is an urgent need for an accurate and automated computer-aided diagnosis for pneumonia detection.

Deep learning is a new technology used in image recognition, natural language processing, speech recognition and other fields. In recent years, deep learning algorithm, especially convolutional neural networks (CNNs) were used in medical image analysis. And achieved remarkable performance in different tasks, such as, medical image classification, lesion segmentation, lesion detection, etc. Specifically, deep learning has been used in brain tumor segmentation [9], breast cancer diagnosis [10], lung nodule detection [11–13], abdominal disease diagnosis [14, 15], and bone disease diagnosis and measurement [16, 17]. Moreover, there has been some studies on pneumonia detection [18–22]. Many studies are used to diagnose COVID-19 pneumonia, and some studies only classify different pneumonias, and cannot use limited information to complete the localization of pneumonia areas.

Therefore, in this study, we developed a DL model for automatic and accurate pneumonia detection using weak supervision information. The model can not only detect pneumonia cases from normal cases, but also localize areas of pneumonia. Furthermore, we evaluated model performance using independently public dataset and performed well.

## Materials and methods

### Data sets

Two separate publicly open and available sources datasets were used for this study [18]. The first public dataset (cohort 1) is from Kermany dataset (https://www.kaggle.com/paultimothymooney/chest-xray-pneumonia) [23]. The dataset includes both pneumonia and normal chest X-ray images, with a total of 5856 images in JPEG format sourced from Guangzhou Women and Children’s Medical Center. These images were obtained as part of routine clinical care for patients. To ensure data quality, all images were initially screened and any low-quality or unreadable scans were removed. Diagnostic results were determined by two expert physicians. Additionally, the second public dataset used in the analysis was sourced from the RAIG dataset (cohort 2). Researchers from Qatar University and the University of Dhaka, along with collaborators from Pakistan and Malaysia and some medical doctors, collated a database of chest X-ray images for COVID-19 positive cases, as well as normal and viral pneumonia images. These chest X-ray images were selected from the database for analysis [24]. There are a total of 3900 X-ray images in cohort 2.

### Deep learning algorithm

We utilized the ResNet-34 deep learning algorithm for pneumonia detection in our study. This algorithm was chosen because it simplifies the training of deeper neural networks. ResNet-34 was selected for its unique architecture, particularly its depth and skip connections. Unlike traditional deep networks, ResNet-34 employs residual blocks that facilitate the training of very deep networks. The skip connections allow the gradient to flow more easily during backpropagation, mitigating the vanishing gradient problem. This makes ResNet-34 well-suited for tasks where capturing intricate features or patterns is crucial, as is often the case in medical image analysis. The ResNet-34 model was pretrained on the ImageNet dataset [25, 27], a large classification dataset, and then fine-tuned for our specific task. This process is commonly known as ‘transfer learning‘ [26]. The final layer of the model was modified to include two neurons, allowing for the distinction between pneumonia cases and normal cases.

**Fig. 1:**
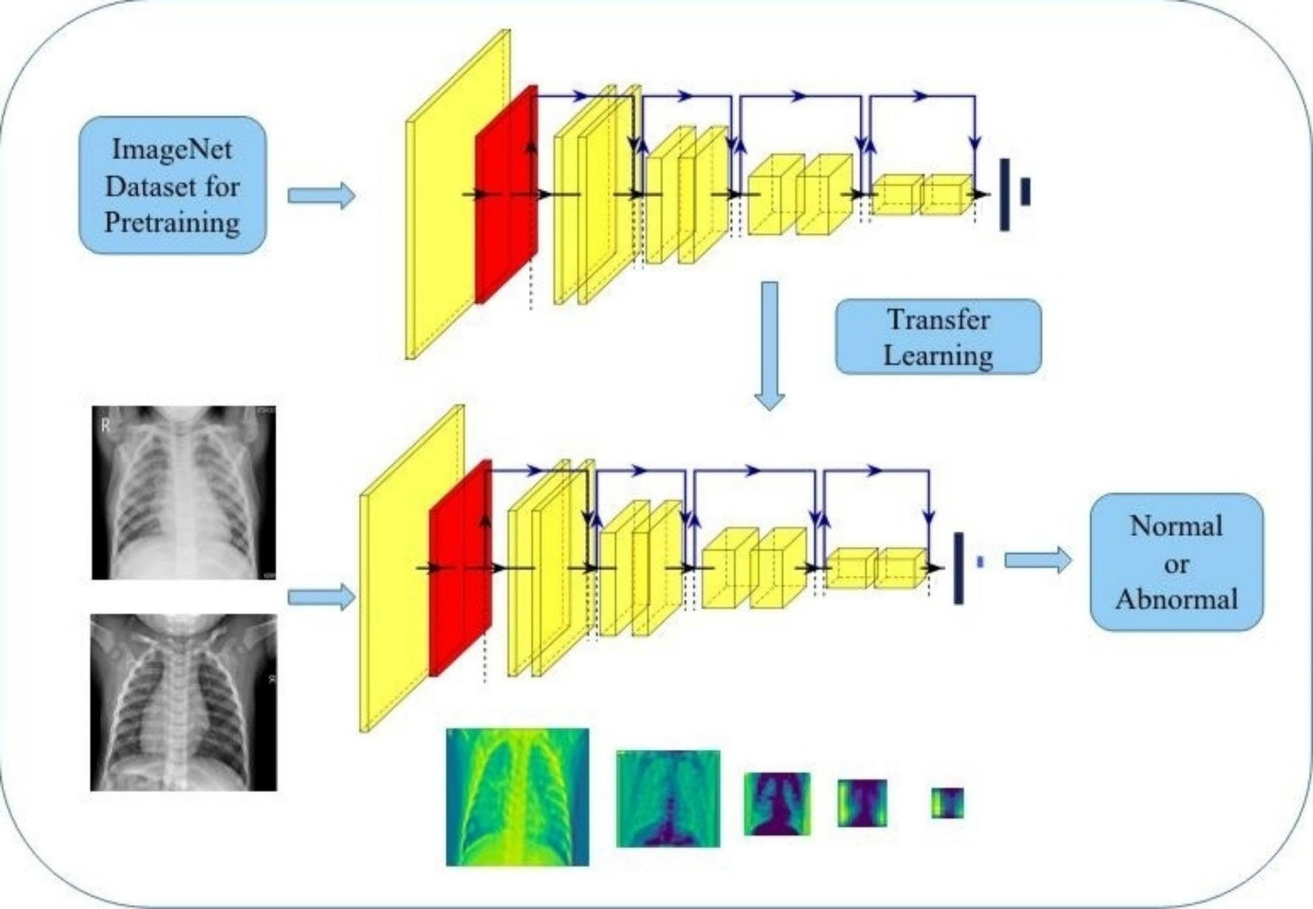
The algorithm flow chart

When the CNN processes the classification task, the ability to locate the pneumonia will be lost due to the fully connected layer. In our study, we use global average pooling (GAP) in CNN for generating CAM. GAP outputs the average of the feature map at the last convolutional layer. The weighted sum of those values is used to generate the final output [27]. Similarly, we compute a weighted sum of the feature maps of the last convolutional layer to obtain CAM. And, the CAM for a particular category indicates the discriminative image regions used by the CNN to identify the pneumonia area. The algorithm flow chart is shown in Fig. 1.

### Training and validation

#### Image processing

The raw images provided by the public dataset are converted to PNG or JPEG format. The images were resized to 224*224. Because deep learning models usually require a lot of data for training. In general, the more the data, the better the performance of the model. Deep learning models cannot learn the pattern or function from the data without enough training data. Therefore, in our study, we used data augmentation technical to increase out data, such as horizontal flip, vertical flip and rotation. And last, before the image enters the network, the pixel values of the images are normalized to [0–1].

#### Training details

The cohort 1 was divided into training set, validation set and test set according to the ratio of 8:1:1. The ResNet-34 model was based on the PyTorch framework and trained on two NVIDIA TITAN XP graphics processing units. Stochastic gradient descent (SGD) with a weight decay of 0.0001 and momentum of 0.9 to optimize the detection model. We train 100 epochs with image batch size 128 on GPU, and the learning rate was set 0.0001. The loss function was classic cross-entropy with softmax. The training process lasted for 19 h. The X-ray images from cohort 2 were enrolled for independent external validation.

### Statistical analysis

Statistical analysis was performed using R software (version 3.5.2, R Foundation for Statistical Computing, Vienna, Austria). The area under curve (AUC) with 95% confidence intervals, accuracy, sensitivity and specificity were selected as performance metrics for the deep learning model. And, in order to compare the detection performance of the model for viral pneumonia, bacterial pneumonia and COVID-19 pneumonia, the identification accuracy was used for evaluation. And proportion test (prop.test() in R) was used for significance test. P < 0.05 indicated statistical significance.

## Results

### Data characteristics

The characteristics of X-ray images that from two public datasets are shown in Table 1. Cohort 1 includes 1493 viral pneumonia cases, 2780 bacterial pneumonia cases, and 1583 normal cases. And cohort 2 (External validation) contains 2600 viral pneumonia cases, including 1300 cases of new coronary pneumonia and 1300 cases of other viral pneumonia. There are 1300 cases in the normal group. And no bacterial pneumonia in cohort 2.

**Table 1:** Characteristics of X-ray images in two cohorts

		Training set	Validation set	Test set	External validation set
Pneumonia	Viral	1195	149	149	1300 (COVID-19) 1300 (Viral)
	Bacterial	2224	278	278	-
Normal	-	1267	158	158	1300
All	-	4686	585	585	3900

### The performance of ResNet-34

**Fig. 2:**
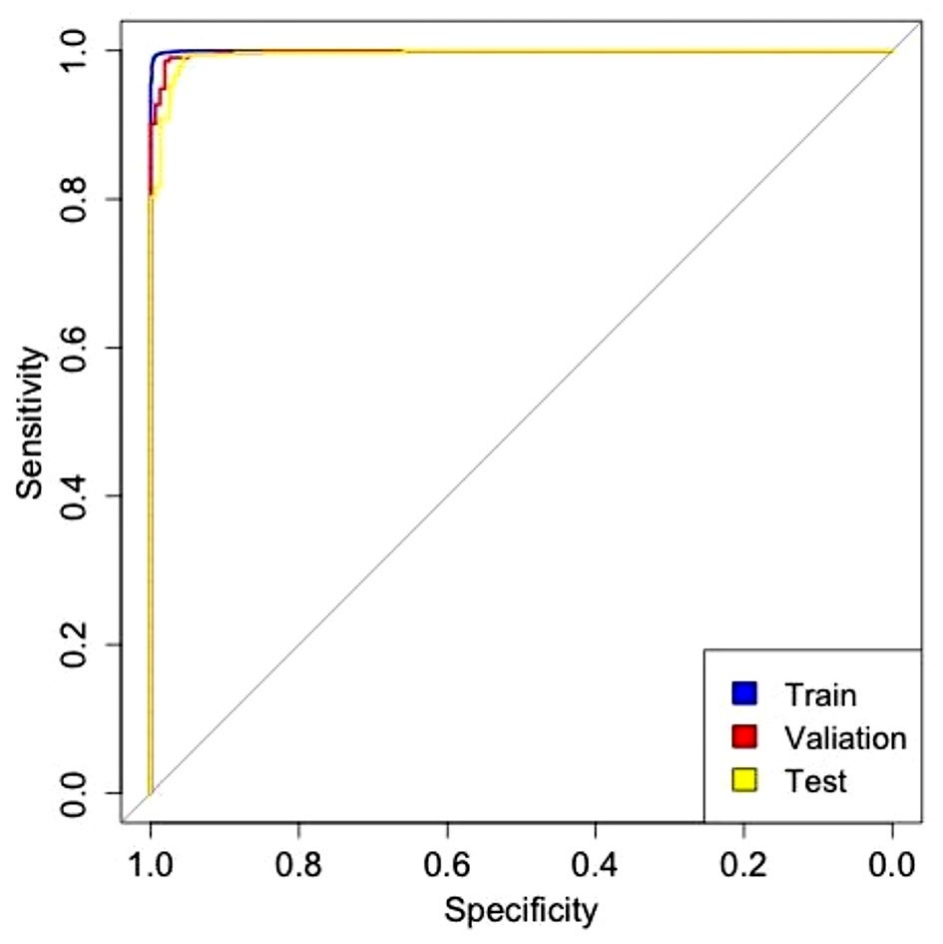
The ROC curve of training set, validation set and test set

The performance results on the chest x-ray image dataset were evaluated on two cohorts. Cohort 1 was divided into training set, validation set and test set. The performance analysis presented in Table 2 shows that our model achieved an AUC of 0.9998 with an accuracy of 99.34% a sensitivity of 99.34% and a specificity of 99.37% in the training dataset. Our model also achieved an AUC of 0.9949 on the test dataset with an accuracy of 98.29%, a sensitivity of 99.29% and a specificity of 95.57% performance. In particular, we collect another publicly available (cohort 2) dataset to evaluate the accuracy and robustness of the model. Finally, the model achieved comparable results on the external test dataset (cohort 2) with an AUC of 0.9835 with an accuracy of 94.62% a sensitivity of 92.35% and a specificity of 99.15%. The model in this study has good robustness and generalization ability to handle different data. The ROC curve of training set, validation set and test set is presented in Fig. 2.

**Table 2:** Diagnostic efficacy of ResNet-34

	AUC	AUC (95%CI)	Accuracy	Sensitivity	Specificity
Training set	0.9998	0.9997–0.9999	99.34%	99.36%	99.37%
Validation set	0.9979	0.9961–0.9993	98.46%	98.59%	98.10%
Test set	0.9949	0.9910–0.9981	98.29%	99.29%	95.57%
External validation set	0.9835	0.9806–0.9864	94.62%	92.35%	99.15%

### Subgroup analysis of the model on the test dataset and external validation dataset

**Fig. 3:**
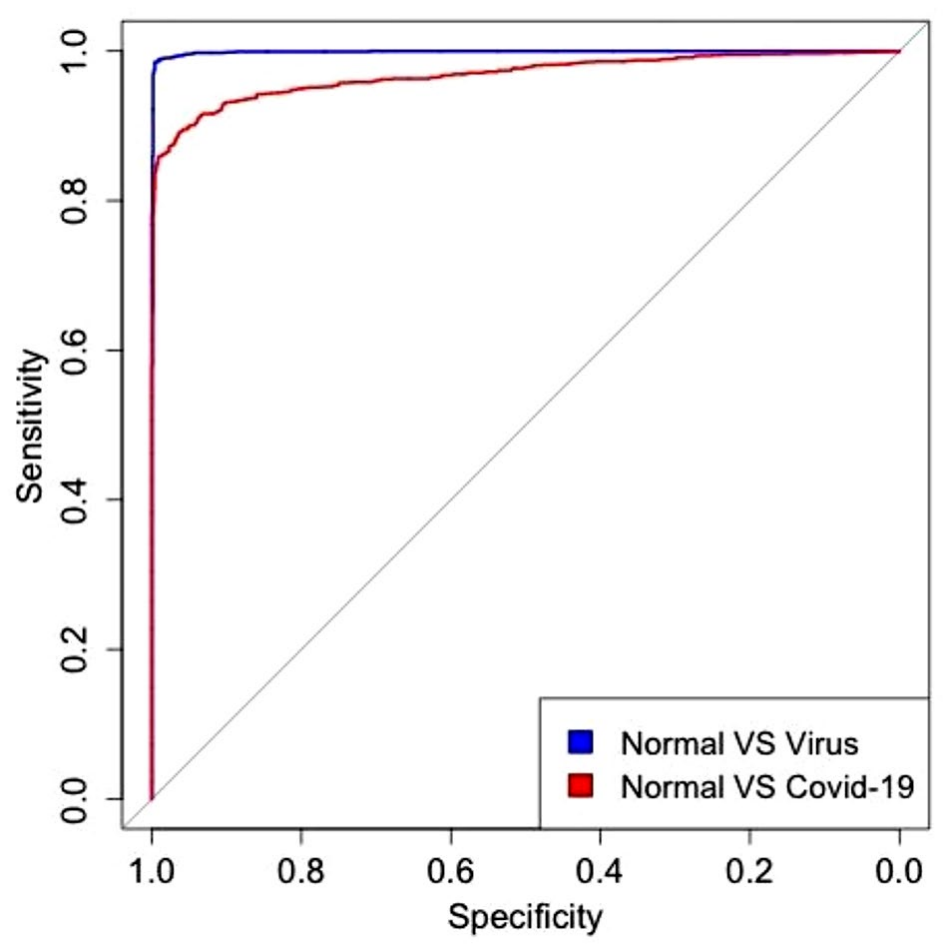
ROC curve for diagnosing of different types of pneumonia

In our study, cohort 1 have different pneumonias, including viral pneumonia and bacterial pneumonia. For cohort 2, although there is no bacterial pneumonia, there is COVID-19 pneumonia and other viral pneumonia. The model can well differentiate between pneumonia cases and normal cases. To further investigate the diagnostic performance for different pneumonias, we performed subgroup analyses of different pneumonias in test dataset and external validation dataset to see if there were differences. Table 3 shows the identification accuracy for different pneumonias. From Table 3 we can find that the identification accuracy of the model for bacterial pneumonia is higher than that for viral pneumonia in the test dataset (98.92%>94.63%, P = 0.0095). And in external validation dataset, the identification accuracy of the model for viral pneumonia is higher than that for COVID-19 pneumonia (98.54%>83.54%, P < 0.0001). The ROC curve is presented in Fig. 3.

**Table 3:** Identification accuracy for different pneumonias

Dataset	Type	Number of correct	Number of wrong	Number	Identification accuracy
Test set	Viral	141	8	149	94.63%
Test set	Bacterial	275	3	278	98.92%
External validation set	Viral	1281	19	1300	98.54%
External validation set	(COVID-19)	1086	214	1300	83.54%

### Localization of Pneumonia regions by weakly supervised algorithms

**Fig. 4:**
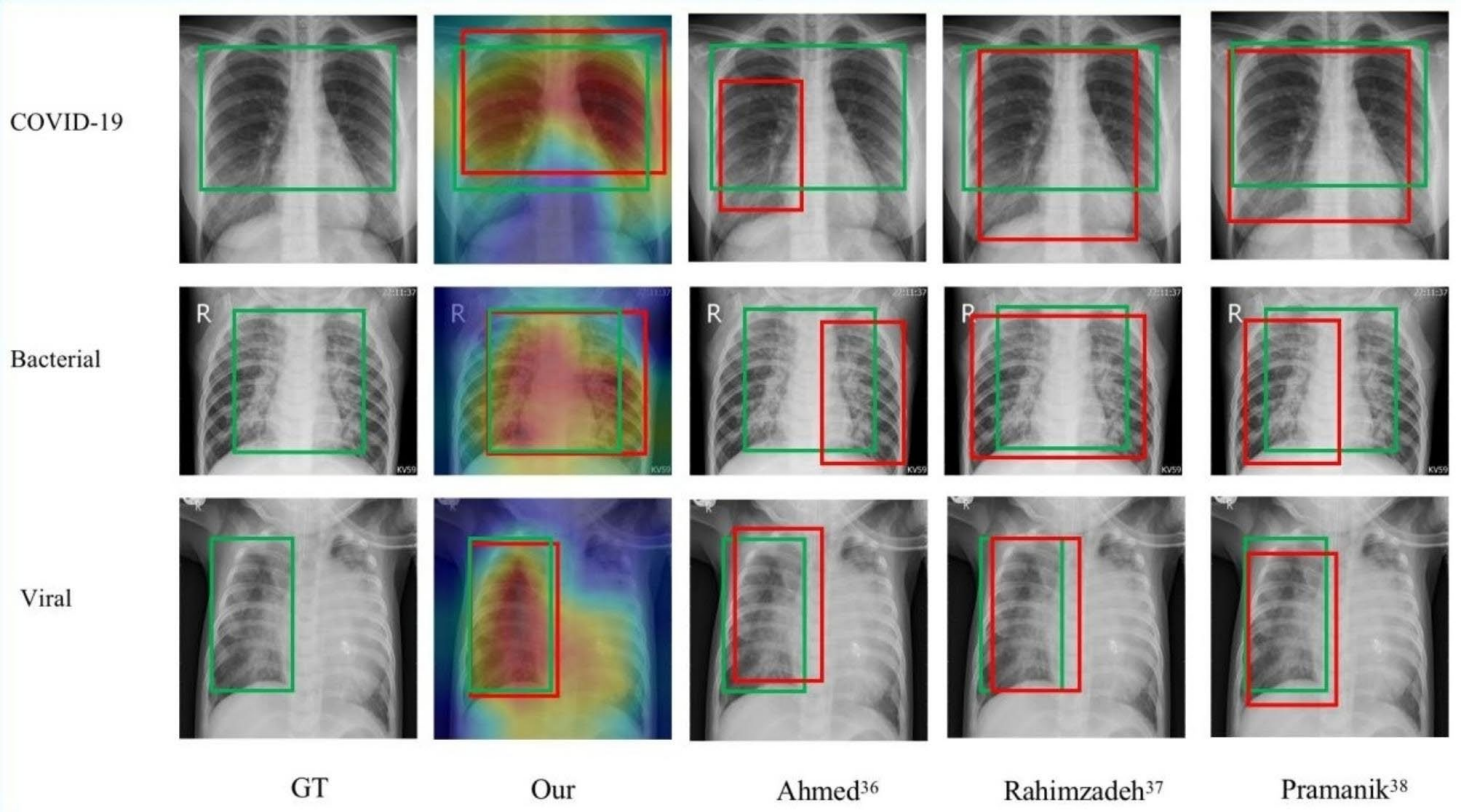
The pneumonia detection result by the CAM method(Green: GT; Red: Predicted)

In our study, we only used image-level labels, i.e. presence or absence of pneumonia, to train the diagnostic model. The localization information of pneumonia was not used when training the model. And, we make used of CAM to understand and clarify the overall impact of pneumonia regions in a given X-ray image as far as diagnostic decisions of the model. Therefore, the pneumonia regions can be located by significant feature areas in the X-ray images. And we randomly selected 6 images in the external test dataset to show the localization accuracy. It can be seen from Fig. 4 that the result of the CAM method shows the ROI of the ResNet-34 model overlaps with the pneumonia area. Meanwhile, We randomly selected 20 images and invited chest radiology experts to annotate the pneumonia area, and then we calculated the Intersection over Union (IoU) of the annotate the pneumonia area and the heat map generated by the CAM. Finally, it was found that the average IoU of the area marked by the doctor and the pneumonia area reached 0.82.

### Comparisons with state-of-the-art methods

In contrast to some previous studies [33–35], the network framework employed in our research is characterized by its lightweight and concise nature, making it easier to implement in clinical practice. Furthermore, our model exhibits superior performance, aiding doctors in making informed clinical decisions. Specifically, we trained the model utilizing a public database, followed by independent external testing on another public database. The external testing results underscored our model’s robustness and generalization capabilities. To better replicate cutting-edge methods, we sought comparisons with open-source code methods. Employing the same data, we thoroughly trained the model and compared it against an external test set. We successfully replicated the methodologies of the three aforementioned studies, with their respective models achieving accuracies of 87.82% [36], 82.33% [37], and 85.79% [38] in the external test data, falling short of our study. Our model’s exceptional performance demonstrates the potential of our proposed model in assisting clinical decision-making processes. In our study we also tried other networks, such as VGGNet, GoogLeNet, MobileNet, DenseNet and EfficientNet. The detailed results are shown in Table 4.

**Table 4:** Identification accuracy for different pneumonias

Method	Accuracy	Sensitivity	Specificity
Ahmed [36]	87.82%	88.31%	86.85%
Rahimzadeh [37]	82.33%	81.12%	84.77%
Pramanik [38]	85.79%	82.50%	92.38%
VGGNet	80.26%	85.42%	69.92%
GoogLeNet	82.59%	88.54%	70.69%
MobileNet	88.82%	90.96%	84.54%
DenseNet	87.13%	89.15%	83.08%
EfficientNet	89.69%	88.85%	91.38%
Our	94.62%	92.35%	99.15%

## Discussion

In this study, we proposed a deep learning weakly supervised algorithm for pneumonia detection. The model we developed can not only distinguish between normal cases and pneumonia cases, but also localize pneumonia regions using image-level information. The model achieved a high AUC of 0.9949, with a accuracy of 98.29%, sensitivity of 99.29% and specificity of 95.87%. And in the cohort 2, we also achieved competitive result with an AUC of 0.9835.

There are already studies on the diagnosis of pneumonia. Rohit Kundu et al. used an ensemble of deep learning methods on the two pneumonia datasets [1]. Many deep learning models are combined for pneumonia detection, so the research method is more complicated and requires higher computing speed and capacity. In our study, we simplified the deep learning algorithm and achieved state-of-the-art results on external datasets. Compared with this study [18], they conducted a differential diagnosis study of different pneumonias, including COVID-19, normal, Viral and Bacterial pneumonia. And many studies have designed different deep learning algorithms for the diagnosis of pneumonia, but using machine learning (ML) methods has several limitations, including complexity, overfitting and poor performance while training with small dataset size [18, 19, 21, 22, 28–32]. Some studies in above studies, the models were not independently validated with external dataset, and some models cannot locate the pneumonia area. Therefore, we proposed a simple deep learning algorithm for pneumonia detection and used image-level weakly supervised information to localize the pneumonia region, and achieved remarkable performance.

Compared with some others studies [33–35], the network framework used in this study is lighter and more concise, more convenient to apply in clinical practice, and the model performance is better, which can assist doctors in making clinical decisions. Specifically, we trained the model using a public database and conducted independent external testing using another public database. The results of external tests illustrate the good robustness and generalization ability of our model. In order to better reproduce the most advanced methods, we found methods with open source code for comparison. We fully trained the model using the same data and compared it on an external test set. We have reproduced the methods of these three studies, and the three models have achieved 87.82% [36], 82.33% [37] and 85.79% [38] accuracy respectively in the external test data, and lower than our study. Our model’s excellent performance illustrates the potential of this study’s model to assist clinical decision-making.

From Table 3, we can see that the identification accuracy of the model for bacterial pneumonia is higher than that for viral pneumonia in the test dataset. The reason for this situation may be due to the fact that there are more bacterial pneumonia cases than viral pneumonia cases in the training data. Since there are more bacterial pneumonia cases than viral pneumonia cases, the deep learning model can better learn the features of bacterial pneumonia, resulting in better identification of bacterial pneumonia cases by the model. The same situation happens with external validation dataset. In the external validation dataset, the identification accuracy of the model for viral pneumonia is higher than that for COVID-19 pneumonia. Patients diagnosed with COVID-19 present symptoms similar to pneumonia. And, some of the findings frequently encountered in COVID-19 pneumonia are: ground glass opacities (GGO), consolidation, crazy paving and enlargement of subsegmental vessels (diameter greater than 3 mm) in areas of GGO [39–43]. It is not completely consistent with other pneumonia manifestations [44]. In the training set, because the model was not trained with COVID-19 data and could not learn the features of COVID-19, so the model had a low identification accuracy for COVID-19. Although the accuracy of the model for the identification of COVID-19 pneumonia is relatively low, it also achieves an accuracy of 83.54%, which has good clinical value for preliminary screening of COVID-19 pneumonia.

Besides, the CAM experiment was conducted to test whether pneumonia regions could be accurately distinguished from other normal regions. After testing, we found that the CAM are quite useful to precisely and accurately locate pneumonia regions in provided X-ray images. Since deep learning is a black box, it cannot be well explained. However, we found that the diagnosis of this study’s model is not incomprehensible and is trustworthy. The model pays more attention to the lesion area than normal area in the pneumonia X-ray images. It also indicates that the model can effectively distinguish the pneumonia area from the none-pneumonia area. It proves that our model is based on the pneumonia region to complete the diagnosis work. At the same time, we can also use image-level information to complete the detection of pneumonia lesions and locate it.

Our study had some limitations. First, the model was only trained and test on public datasets, and no further validation was performed on data from actual hospital institutions. Second, there is no bacterial pneumonia in the external validation dataset, so the model cannot be further validated for the diagnostic performance of bacterial pneumonia. For the above two limitations, in future study, we will collect data from our hospital to further validate the model’s performance. Third, we did not perform quantitative analysis. Only some images were randomly selected to show whether the model detected pneumonia is accurate. Therefore, in future research, we will compare whether the pneumonia area marked by the doctor is consistent with the pneumonia area identified by the model. Fourth, the method we proposed is to use the threshold set by the result of the CAM, which can obtain the specific location of pneumonia. However, this threshold is fixed, so there may be some deviations in locating pneumonia regions in some images. In our subsequent studies, we plan to make this threshold a learnable parameter for the model to increase the accuracy of localization.

In conclusion, we proposed a deep learning algorithm can accurately detect pneumonia and locate the pneumonia area based on weak supervision information, which can provide potential value for helping radiologists to improve their accuracy of detection pneumonia patients through X-ray images.

## Data Availability

The data used in this paper is from two publicly available dataset. The first dataset can be accessed at https://www.kaggle.com/paultimothymooney/chest-xray-pneumonia and the second can be accessed at https://figshare.com/articles/dataset/RAIG_dataset_zip/14151854/4. This is the third party material we have used in this study.

## Abbreviations

DL: Deep learning
CAM: Class activation map
CNN: Convolutional Neural Network
AUC: area under the curve
ROC: receiver operating characteristic curve
ResNet34: residual network with 34 layers
COVID-19: coronavirus disease 2019
95%CI: 95% confidence interval
CT: Computed Tomography
